# Long-term outcome of low-activity radioiodine administration preceded by adjuvant recombinant human TSH pretreatment in elderly subjects with multinodular goiter

**DOI:** 10.1186/1756-6614-2-6

**Published:** 2009-06-30

**Authors:** Massimo Giusti, Mauro Caputo, Iolanda Calamia, Mariaclaudia Bagnara, Enrica Ceresola, Mara Schiavo, Michele Mussap, Diego Ferone, Francesco Minuto, Marcello Bagnasco

**Affiliations:** 1 Clinica Endocrinologica, Azienda Ospedaliera Universitaria "San Martino", Genoa, Italy; 2 Terapia Radiometabolica, Azienda Ospedaliera Universitaria "San Martino", Genoa, Italy; 3 Fisica Sanitaria, Azienda Ospedaliera Universitaria "San Martino", Genoa, Italy; 4 Laboratorio Analisi, Azienda Ospedaliera Universitaria San Martino, Genoa, Italy

## Abstract

**Background:**

Large multinodular goiter (MNG) in elderly people is a common finding which can require intervention. The long-term effect of radioiodine therapy on thyroid volume (TV) and function after recombinant human (rh) TSH pre-treatment was evaluated.

**Methods:**

After baseline evaluation, 40 subjects over 60 years old with a large MNG were treated with ^131^I up to the activity of 600 MBq. Nineteen patients were pretreated with rhTSH (0.1 mg on 2 consecutive days; group 1) while 21 subjects underwent treatment without rhTSH pretreatment (group 2). TV was monitored every 6–12 months by ultrasonography. The median follow-up period was 36 months.

**Results:**

At the baseline, the groups matched in terms of TV, 24-h radioiodine uptake (RAIU), urinary iodine and neck complaints. The number of subjects pretreated with anti-thyroid drugs was significantly (P = 0.01) greater in group 2 than in group 1; TSH was more suppressed (P = 0.003) and f-T3 was more elevated (P = 0.005) in group 2 than in group 1 patients. RhTSH increased 24-h RAIU in group 1 up to the baseline level observed in group 2. The ^131^I activity administered was similar in both groups. Adverse events were slight and similar in both groups. A permanent post-radioiodine toxic condition was reported only in 2 patients in group 2. After radioiodine therapy, hypothyroidism was observed in significantly more group 1 patients than group 2 patients (P = 0.002). While TV was reduced in both groups, the percentage TV reduction recorded at the last examination was significantly higher (P = 0.03) in group 1 than in group 2. MNG-related complaints were significantly reduced in both group 1 (P = 0.0001 vs baseline) and group 2 (P = 0.001) patients.

**Conclusion:**

Low radioiodine activities after pretreatment with low-dosage rhTSH are able to reduce TV and improve MNG-related symptoms in elderly subjects.

## Background

Italy is an area of moderate iodine deficiency, where goiter is rather common. Goiter due to iodine deficiency progresses from a diffuse enlargement of the thyroid in young adults to a multinodular structure with advancing age [1]. Patients with large multinodular goiter (MNG) frequently complain of signs and symptoms that range from a slight sense of constriction to more severe compressive disorders and impaired respiration [1]. While the functional activity of MNG is variable, in elderly subjects TSH sometimes declines as a consequence of autonomous hormonal activity of the nodules, with clinical features ranging from euthyroidism to sub-clinical or overt hyperthyroidism. In these patients, surgery is the standard procedure but it may be contraindicated because of cardiac, pulmonary, or other chronic disorders. When surgery is not practicable or is refused by the patient, radioiodine (RAI) therapy may be used to reduce thyroid volume (TV) in enlarged MNG [2-5]; however, the rather low RAI uptake (RAIU) and the need for intense RAI activity can make this therapeutic procedure less effective.

Recombinant human TSH (rhTSH) is now used in the diagnosis and therapy of differentiated thyroid cancer, and may be of value in some other conditions [6,7]. One instance is the use of rhTSH to increase RAIU in patients with benign MNG, as recently reported in a review by Fast et al. [8]. RhTSH pre-treatment has been shown to increase thyroid RAIU and to modify the regional distribution of RAI by stimulating RAIU in hypofunctioning regions within a goiter [9]. Huysmans et al. [10] showed that a single 0.01–0.03 mg rhTSH dose administered 24-h before a diagnostic RAI dose was able to double RAIU in patients with MNG. Subsequently, it was shown that two 0.1 mg doses of rhTSH administered on two successive days increased the mean RAIU 4.5-fold, a result that closely parallels the reported effectiveness of a single 0.3 mg dose [11]. Several observational and prospective controlled studies in the last 4 years have demonstrated that rhTSH combined with RAI treatment can reduce TV by up to 57% [11-19].

Current literature data seem to indicate that using rhTSH as an adjuvant to RAI therapy is more effective than using RAI alone, when the same radioiodine activities are compared. When used for the purpose of calculating the activity of the RAI dose administered, rhTSH allows lower amounts of radiation to be used. However, the optimal rhTSH dose and the minimum effective RAI activity needed to treat non-toxic MNG remain to be established. In addition, the absence of a correlation between TV reduction and post-rhTSH RAIU, area under the curve of TSH after rhTSH, baseline TV or RAI activity actually exerted suggests that goiter reduction may depend on other factors related to rhTSH pre-stimulation, and not only on the RAI activity applied [7]. Finally, it should be borne in mind that treatment with rhTSH and RAI has many undesirable consequences, such as transient hyperthyroidism, thyroid pain and acute goiter swelling [6].

In Italy, the maximum RAI activity allowed for therapy in out-patients is 600 MBq. In a previous study [16] we assessed 20 elderly patients with large goiters and compared treatment with RAI activities (370–555 MBq, fixed activity) following two consecutive 0.2 mg doses of rhTSH with treatment with ^131^I alone. In comparison with patients who were treated with ^131^I alone, those who received rhTSH had higher transient (lasting 2 weeks) elevation of thyroid hormones, a greater reduction in TV, and a similar incidence of hypothyroidism about 2 years after therapy [16].

In the present study, we report the long-term outcome of a larger group of elderly out-patients with large MNG treated with weighted ^131^I activities up to 600 MBq after pre-treatment with two consecutive 0.1 mg rhTSH doses. To assess safety, TV reduction and the duration of thyroid function, the results were compared with those observed in a group of out-patients matched for age and TV in whom MNG was conventionally treated with radioiodine up to 600 MBq.

## Methods

### Study population and design

We enrolled 49 patients over 60 years of age with a long history of MNG and symptoms of cervical compression and esthetic discomfort; in all patients, cytology after fine-needle aspiration biopsy of suspect nodules was non-malignant and neck surgery was refused or not feasible, owing to other severe concomitant diseases. All patients lived in Liguria or southern Piedmont, Italian regions where iodine prophylaxis is recommended on account of mild-to-moderate iodine deficiency [20]. The administration of anti-inflammatory steroids and beta-blockers was not contraindicated in any patients, all of whom were on these drugs when the protocol started.

Twenty-three out-patients (age range 60–84 yr) attending our Endocrine Unit were selected for RAI therapy after rhTSH pre-treatment (group 1). While thyroid hormones were normal in the majority of patients, some patients displayed a pre-toxic condition (TSH suppressed) on baseline evaluation. Four of the 23 patients were excluded from the study on account of high iodine excretion, elevated baseline RAIU, or the need for RAI activity greater than that allowed for out-patients (n = 2). Table 1 reports some clinical data on the 19 group 1 patients (age range 60–84 yr) who completed the protocol. Forty-eight and 24-h before the administration of RAI therapy, patients received 0.1 mg of rhTSH, which was obtained from freeze-dried vials containing 0.9 mg of the drug (Genzyme, Cambridge, MA, USA), reconstituted with 1 ml of sterile water and then diluted to a final concentration of 0.1 mg/ml with normal saline.

**Table 1:** Baseline clinical and laboratory data of outpatients with MNG treated with RAI with (group 1) or without (group 2) rhTSH pre-treatment (0.1 mg, on two consecutive days).

	**Group 1 (n = 19)**	**Group 2 (n = 21)**	**Significance**
Age (yr), mean ± SD	71.8 ± 6.7	70.6 ± 6.8	ns
Females/Males	18/1	16/5	ns
BMI (kg/m^2^)	27.6 ± 1.1	26.4 ± 0.7	ns
Previous surgery (n)	3	3	ns
Previous ^131^I therapy (n)	2	2	ns
Use of MMI before RAI (n)	5	14	P = 0.01
US TV (ml), mean ± SEM	71.9 ± 8.1	79.5 ± 8.1	ns
(median; range)	65.0; 39 – 171	72; 38–194	
TSH (mIU/l), mean ± SEM	0.69 ± 0.11	0.50 ± 0.24	P = 0.003
f-T3 (pmol/l), mean ± SEM	5.1 ± 0.3	5.7 ± 0.1	P = 0.005
f-T4 (pmol/l), mean ± SEM	16.2 ± 0.8	16.6 ± 1.0	ns
Tg (μg/l), mean ± SEM	93.5 ± 21.8	81.5 ± 14.6	ns
Positive thyroid antibodies (n)	4	2	ns
Urinary iodine (μg/l), mean ± SEM	97.2 ± 9.8	79.6 ± 12.9	ns
24-h RAIU (%), mean ± SEM	35.2 ± 3.2	43.4 ± 2.9	ns
^131 ^I activity (MBq), mean± SEM	542.1 ± 18.3	549 ± 13.8	ns
(median; range)	600; 370 – 600	555; 370 – 600	

Twenty-six out-patients (age range 60–82 yr) attending our Radiometabolic Therapy Unit were selected for RAI treatment without rhTSH pre-treatment (group 2). In this control group, the majority of patients were in a pre-toxic condition, but in some cases hyperthyroidism was found on baseline evaluation. Five of these patients were excluded from the study on account of the need for greater RAI activity than that allowed for out-patients (n = 1), low RAIU uptake after methimazole (MMI) discontinuation (n = 2), and patient refusal (n = 2). Some clinical data on the 21 group 2 patients (age range 60–82 yr) who completed the protocol are reported in Table 1.

All patients who were on MMI at the baseline suspended the drug 3 – 4 weeks before initial thyroid volume (TV) and 24-h RAIU evaluations and further RAI treatment.

The study was approved by the local ethics committee, and all patients provided signed informed consent.

### Uptake measurements and RAI therapy

RAI therapy was dosed according to TV and RAIU, up to the maximum allowed dose of 600 MBq for out-patients in Italy. The individual RAI doses administered were calculated according to the method of Traino et al. [21]. For each patient, iodine pollution was excluded in advance by the evaluation of 24-h urinary iodine excretion. Thyroid ^131^I uptake was determined 24 h after oral administration of a tracer activity of 0.5 MBq ^131^I. In group 1 patients, RAIU evaluation was repeated under rhTSH administration, just before RAI therapy. From day 0 to day 7 of RAI therapy, all subjects received a 25 mg oral dose of atenolol and a 4 mg intramuscular dose of betamethasone daily.

### Thyroid size assessment

TV was assessed in all patients by means of ultrasonography (US) before ^131^I treatment, and, in the vast majority, 6, 12 and 24 months after treatment and at irregular intervals thereafter. US was performed on two-dimensional images by the same operator (MG) and further validated independently by a second experienced physician by means of an Esaote AU5 Idea in combination with a 7.5 MHz linear transducer (Esaote, Genoa, Italy). Subjects lay supine on the examination bed with the neck hyper-extended. The thyroid lobes were scanned separately. Assuming that both lobes are geometrically shaped like rotated ellipsoids, TV was determined by applying the formula width × depth × length × π/6. Width and depth dimensions were measured in the transverse scan in terms of the maximum cross-sectional area of the thyroid lobe. Length was measured in the longitudinal scan of the thyroid lobe. When thyroid dimensions were larger than the field of view of the ultrasound beam, an extended field-of-view ultrasound or dual-image (split-screen) ultrasound technique was used. When an isthmus of measurable size (> 1 ml) was found, its volume was calculated accordingly. All measurements (mm) were obtained by using the built-in electronic calipers. The sum of the volumes of the right lobe, left lobe and isthmus was regarded as the total TV. In patients who had previously undergone thyroid surgery, TV was calculated on the residual thyroid gland. Coefficients of variation of repeated US investigations were less than 20% [16,22].

### Laboratory evaluations

Blood samples for functional thyroid studies were collected at each examination before ^131^I treatment and during the prolonged follow-up period. Serum TSH, serum free-T3 (f-T3) and free-T4 (f-T4), serum Tg, thyroid peroxidase antibodies (TPOAb) and Tg antibodies (TgAb) were evaluated. TSH, f-T3 and f-T4 were measured by means of ultra-sensitive chemiluminescence immunoassay (Roche Diagnostics, Mannheim, Germany). Normal ranges are 0.3–4.2 mIU/l for TSH, and 3.9–6.8 pmol/l and 12.0–22.0 pmol/l for f-T3 and f-T4, respectively. Tg was assayed by chemiluminescence immunoassay (Roche Diagnostics); the normal Tg value in patients without goiter is <90 μg/l. TPOAb and TgAb were measured by DiaSorin assays (Saluggia, Italy); concentrations < 50 and <100 mIU/l, respectively, were regarded as negative. Urinary iodine concentrations were estimated by means of a calorimetric analytical method (Ioduria Kit, Cell-tech, Turin, Italy) on spot urine specimens, and were expressed as μg/l.

### Statistical analysis

The primary objective of the study was to evaluate TV after treatments. Secondary objectives were to evaluate changes in both subjective symptoms and thyroid function. The severity of acute (first month after treatment) adverse events was carefully evaluated. The subjective benefit on goiter-related symptoms was evaluated by comparing the patient's assessment of subjective discomfort before treatment with that recorded at the end of the follow-up period by means of a visual analogical scale (VAS) ranging from 0 (no complaints) to 10 (subjective maximum degree of cervical discomfort).

Data were analyzed by means of the Prism 4.0 software (GraphPad Software, San Diego, CA, USA). All values quoted are means ± SEM, unless otherwise stated. Significance was taken as P < 0.05. To compare continuous or percentage data, the Kruskal-Wallis analysis of variance was used, followed by Dunn's Multiple comparison test, the Mann-Whitney test, the Wilcoxon test and Fisher's exact test, when appropriate. Correlation analyses between variables were carried out by the Spearman correlation test.

## Results

### Baseline clinical and laboratory data

Table 1 reports the baseline clinical and laboratory data of patients who underwent RAI treatment with (group 1) or without (group 2) rhTSH (0.1 mg on two consecutive days) pre-treatment. The majority of patients treated were females, but no significant difference emerged between the groups in terms of female/male distribution. The variables age, BMI, previous thyroid surgery, urinary iodine concentration, and positive thyroid autoimmunity were similar in both groups. Before RAI therapy, patients with concomitant hyperthyroidism underwent MMI treatment in order to prevent overt thyroid hyperfunction; group 2 contained more of these patients than group 1 (14 vs 5) (P = 0.01). At the baseline, f-T3 levels were slightly but significantly higher (P = 0.005) in group 2 than in group 1, while TSH was lower (P = 0.003). TV and % 24-h RAIU were similar in both groups. In group 1 patients, RAIU increased to 45.0 ± 4.1% following rhTSH pre-stimulation (P = 0.01). The median activity applied was 600 MBq in group 1 and 555 MBq in group 2 (Table 1). In accordance with national regulations for out-patients, ^131^I activity was restricted to 600 MBq in 11 group 1 patients (58%) and 8 group 2 patients (30%; ns *vs *group 1), although the optimal activity would have been higher.

### Thyroid volume reduction

**Figure 1:**
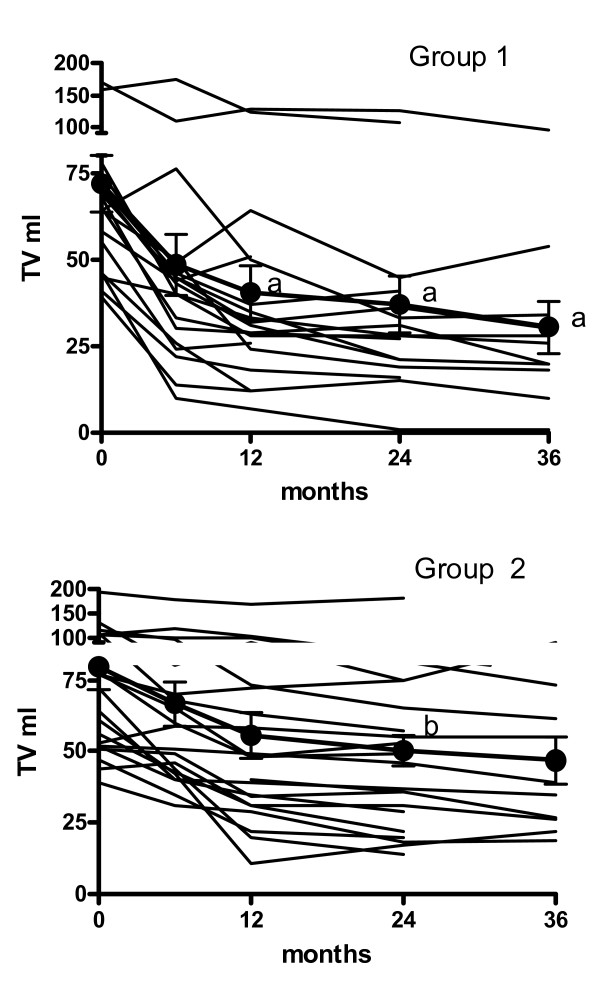
**Individual TV observed over 36 months of follow-up in group 1 (upper panel) and group 2 (lower panel) patients**. Mean ± SEM data are also shown (closed circles). Significances by Dunn's multiple comparison test vs baseline are: a P < 0.01, b P < 0.05.

**Figure 2:**
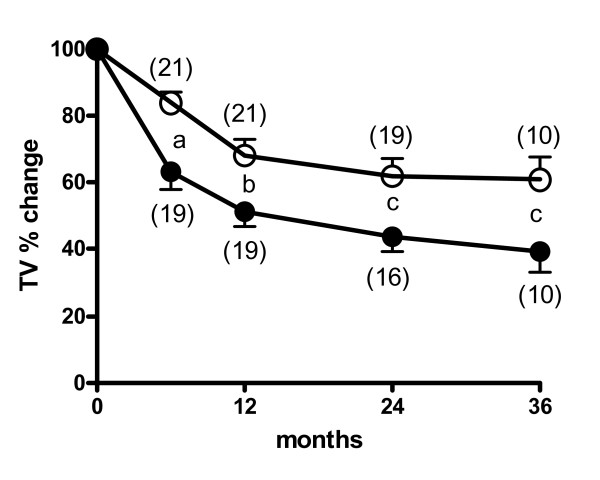
**Change in TV over 36 months of follow-up in group 1 (closed circles) and group 2 (open circles)**. TV at the various time-points is expressed as a percentage (mean ± SEM) of the baseline value. Significances between groups are: a P = 0.001, b P = 0.01, c P = 0.03. The number of patients is shown in brackets.

At the baseline, the median TV was 71.9 ± 8.1 ml in group 1 and 79.5 ± 8.1 ml in group 2 (Table 1). Four patients from group 2 dropped out during the follow-up period: 3 underwent further RAI therapy (TV reduction 3%, 11%, and 31% from baseline) after 24 months, and 1 underwent thyroid surgery (TV reduction 30%) after 42 months. Figure 1 shows the individual and the mean (± SEM) time-course trends of TV in each group. Kruskal-Wallis analysis of variance revealed that TV reduction was greater in group 1 (P < 0.0001) than in group 2 (P = 0.01). Dunn's multiple comparison test revealed no further significant TV reduction from the 6^th ^to the 36^th ^month of follow-up in either group of patients (Figure 1). The average follow-up was 33.3 ± 3.2 months (median 36 months; range 12 – 60 months) in group 1 and 36.9 ± 3.4 months (median 36 months; range 12 – 63 months) in group 2 (ns). At the last evaluation, TV was 29.5 ± 5.2 ml (median 25 ml; range 1–107 ml) in group 1 (P < 0.0001 vs baseline) and 47.5 ± 8.1 ml (median 40 ml; range 10–172 ml) in group 2 (P < 0.0001 vs baseline) patients. At this evaluation, the difference in TV between the groups was near to statistical significance (P = 0.06). Figure 2 reports the mean percentage (± SEM) change in TV following RAI in both groups of subjects. The TV reduction was more evident in group 1 than in group 2 patients. At the last evaluation, the TV reduction was significantly (P = 0.03) greater in group 1 (60.2 ± 4.4%; median 62%, range 12% – 98%) than in group 2 (44 ± 5.4%; median 47%, range 0% – 84%). In both groups, TV reduction was unrelated to age, BMI, initial TV, thyroid function, baseline RAIU or RAI activity.

### Thyroid function and autoimmunity

In the first 4 weeks after RAI treatment, thyroid function was routinely evaluated. No difference in f-T3 (group 1: 5.9 ± 0.5 pmol/l; group 2: 5.7 ± 0.3 pmol/l), f-T4 (group 1: 19.3 ± 1.9 pmol/l; group 2: 18.6 ± 2.3 pmol/l) or TSH (group 1: 0.25 ± 0.19 mIU/l; group 2: 0.57 ± 0.19 mIU/l; P = 0.1) was found 4 weeks after RAI treatment. According to the results of this evaluation, patients were treated or not with MMI. There was no difference between the groups in the percentage of patients on MMI soon after RAI (P = 0.1 by Fisher's exact test). MMI was restarted for 3–12 months in 4 out of 5 group 1 patients previously treated with MMI. In group 2, MMI was restarted in 6 patients previously treated with MMI and in 1 other patient; 5 patients were treated for 3–12 months and 2 for 24 months. Only in group 1 patients (n = 7) did permanent hypothyroidism develop within the first year after RAI; in these patients, L-T4 was started. At the last examination, the percentage of patients on L-T4 was significantly higher (P = 0.002) in group 1 than in group 2 (Table 2). Table 2 reports data on thyroid function, thyroid autoimmunity and ongoing thyroid treatment at the last examination in groups 1 and 2. In all patients, thyroid function was in the normal range at the last examination, both in those on L-T4 or MMI and in those off thyroid drugs. Moreover, f-T3 levels were still slightly, but significantly (P = 0.001), higher in group 2 than in group 1 (Table 2).

**Table 2:** Laboratory data and numbers of patients on therapy to normalize thyroid parameters in MNG out-patients treated with RAI with (group 1) or without (group 2) rhTSH pre-treatment (0.1 mg, on two consecutive days).

	**Group 1**	**Group 2**	**Significance**
TSH (mIU/l)	1.61 ± 0.19	1.36 ± 0.19	ns
f-T3 (pmol/l)	4.0 ± 0.1	4.6 ± 0.1	P = 0.001
f-T4 (pmol/l)	15.3 ± 0.5	14.7 ± 0.6	ns
Tg (μg/l)	168.3 ± 50.4	337.7 ± 268.7	ns
Positive thyroid antibodies (n)	5	3	ns
L-T4 treatment (n)	12	3	P = 0.002
MMI treatment (n)	0	2	ns

### Subjective complaints

No adverse events were reported by 4 group 1 subjects and 6 group 2 subjects in the first 4 weeks after RAI therapy. The complaints reported by the other subjects in both groups were generally short-lived. The most frequent complaint in both groups was "neck trouble". Two patients were hospitalized after RAI: one from group 1 on day 7 owing to hyperkinetic arrhythmia and one from group 2 on day 6 owing to respiratory insufficiency due to concomitant chronic obstructive pulmonary disease.

**Figure 3:**
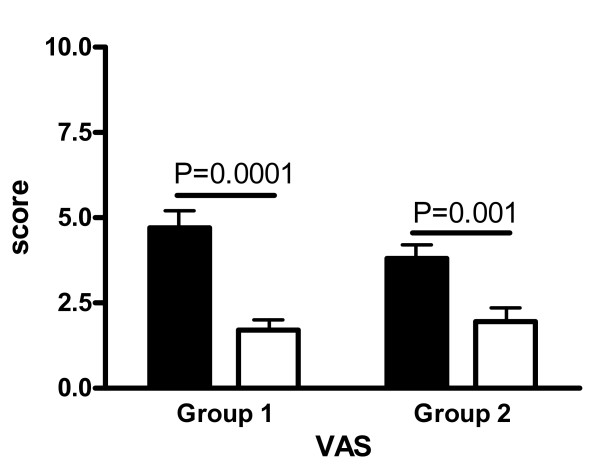
**Mean (± SEM) scores self-reported by patients by means of an *ad hoc *VAS scale administered to all patients at the baseline (closed bars) and at the last examination (open bars)**.

Figure 3 reports mean VAS scores on enrolment and at the last examination. At the baseline, no difference in VAS scores was noted between the groups. In both groups, baseline VAS scores were unrelated to TV. At the last examination, VAS scores were significantly reduced in both groups of patients but the reduction from the baseline was more significant in group 1 (P = 0.0001) than in group 2 (P = 0.001) patients (Figure 3). No change (n = 2) or a slight reduction (n = 2) in VAS score was noted in the 4 group 2 patients who dropped out of the study 24 months after RAI in order to undergo further RAI treatment or thyroidectomy.

## Discussion

While surgery remains the gold standard in the treatment of MNG, several recent studies have demonstrated that RAI therapy with adjuvant rhTSH pre-treatment can be an attractive alternative [11-19]. The prevalence of MNG rises in the elderly, who also often suffer from comorbities [23]. Although many patients enrolled in the several observational, prospective and controlled studies were more than 60 years old, few studies involving this new therapeutic protocol have so far focused on elderly subjects [15,16].

In the present study we prospectively evaluated 40 outpatients with a mean age of 71 years. Within the limits of our protocol (the patients were consecutively recruited without randomization, and rhTSH administration was not blinded/placebo-controlled) the results obtained documented a greater reduction in TV when rhTSH treatment preceded RAI therapy than when similar RAI activities were applied alone. A significant, effective and permanent TV reduction is the main outcome of RAI therapy for MNG in the elderly. In our previous study [16], fixed doses of RAI (370 to 555 MBq) alleviated symptoms in all but one patient following rhTSH administration (0.2 mg on two consecutive days), and yielded a mean 50% reduction in TV after about 2 years. In the present study, TV reduction was greater in the rhTSH-treated group 6–12 months after RAI therapy; thereafter, a slow decline in TV was maintained. On long-term follow-up (median 36 months) evaluation, a 62% reduction was found, which was 15% greater than after RAI alone. So far, rhTSH-pre-treated patients have not required any further treatment to reduce TV, while some patients treated with RAI alone have required further RAI therapy or surgery.

It should be noted that the sample of patients we enrolled comprised both patients with euthyroid MNG and patients with MNG and subclinical hyperthyroidism, as in other series [11,14,16,18,19]). More patients in group 2 (no rhTSH pretreatment) had subclinical hyperthyroidism; consequently, more group 2 patients were treated with low-dose MMI before RAI. This constitutes a potential bias of the study. However, in our opinion, the results of RAI are fairly comparable between the 2 groups, bearing in mind that baseline RAIU and TV were very similar.

It is not easy to compare the data from the various studies, owing to differences in sample populations and therapeutic protocols (different amounts of rhTSH from 0.03 to 0.45 mg; different RAI doses from 370 to 5550). However, Cohen et al. [14] reported a 30% reduction in TV after a 6-month follow-up, which is roughly comparable to that found in our patients. We observed a 50% reduction in TV after 12 months, which is similar to that seen in other studies [11,14,18,19].

It is not clear how long rhTSH and RAI exert their reducing effect on TV. In a follow-up study lasting up to 12 months, Paz-Filho et al. [24] reported an average 50% reduction in TV after RAI (1110 MBq) in patients with an initial TV of 100 ml pre-treated with rhTSH (0.1 or 0.2 mg); most (about 70%) of this reduction occurred in the 1^st ^year after treatment. On the other hand, Cardia et al. [25] reported data from a 3-year follow-up of patients involved in a previously published study [14]. At the last evaluation of available patients, ongoing TV reduction was observed from the 1^st ^to the 4^th ^year after RAI therapy; moreover, the initial difference in TV reduction in favor of rhTSH-pre-treated patients persisted (on average, 70% with rhTSH plus RAI vs 60% with RAI alone [25]. The trend in our data suggests that a similar percentage reduction in TV can be achieved by using lower RAI activities (up to 600 MBq instead of up to 5550 MBq). On the whole, we think that the expected degree of final TV reduction after rhTSH plus RAI is in any case greater than that observed in long-term follow-up studies after RAI alone [3-5].

Forecasting TV reduction is an unresolved issue. We did not find any correlation between TV reduction and age, BMI, initial TV, thyroid function, baseline RAIU or the RAI dose administered. Similar results have been reported by Paz-Filho et al. [19]. According to Bonnema et al. [18], TV reduction may be related to initial TV if the goiter is very large, which is the most frequent situation in elderly patients eligible for rhTSH plus RAI therapy. However, they found that the reduction in the volume of the goiter after rhTSH and RAI was unrelated to the thyroid dose retained, an observation in line with other studies conducted on patients with smaller goiters [17]. Our data, which were obtained at low RAI activity, suggest that the effect of rhTSH depends on factors independent of thyroid irradiation. It is plausible that rhTSH might induce the reactivation of dormant thyroid tissue, or render thyrocytes more vulnerable to ionizing radiation [18]. Indeed, the existence of such preconditioning by TSH has been suggested in studies of hyperthyroid patients treated with RAI [26].

Hypothyroidism and the need for L-T4 treatment might be seen in our protocol as an unwelcome effect of rhTSH pre-treatment. During follow-up, we observed hypothyroidism in 63% of rhTSH-pre-treated patients, while L-T4 treatment was necessary in only 14% of patients treated with RAI alone. This difference could be explained by a difference in functional status between the two groups on enrolment (more thyroid hyperfunction in the group treated with RAI alone), while a different autoimmune response is not proven. Moreover, the efficacy of RAI treatment in MNG is usually hampered by the low and irregular RAIU [1], and it has been shown that rhTSH increases RAIU ([9], [13]), thereby enhancing the adsorbed activity [27].

In subjects (mean age 63 years) with large MNG (59% toxic MNG) treated with high rhTSH (0.45 mg) and RAI (up to 5550 MBq) dosages, Silva et al. [14] observed hypothyroidism in 35% of subjects one year after therapy, as against only 21% (70% toxic MNG) of subjects treated with RAI alone. In another study, Cohen et al. [15] investigated 17 patients (aged >60 years) with a large percentage of toxic MNG (70% of cases); six months after very low rhTSH doses (0.03 mg) administered 24-h before 1100 MBq of RAI, hyperthyroidism was seen to have disappeared in 82% of cases and hypothyroidism in only 18%. In a study by Paz-Filho et al. [19] of 17 subjects (aged >60 years) with normal (n = 12) or increased (n = 5) thyroid function on enrolment, 53% of subjects displayed hypothyroidism 1–2 years after 0.1 rhTSH pre-treatment and 1100 MBq RAI therapy. More recently, Paz-Filho et al. [24] reported data on two cohorts of patients pre-treated (0.2 mg) or not with rhTSH before RAI (1100 MBq); on 2-year follow-up examination, they found a higher prevalence of hypothyroidism in the pretreated (73%) than in the non-pretreated group (53%).

Rubio et al. [28] have claimed that TPOAb positivity may occur after RAI, regardless of rhTSH pretreatment, and Paz-Filho et al. [19] have suggested that TPOAb positivity and the presence of smaller goiters may increase the risk of developing hypothyroidism. Our data do not support these hypotheses, since only a few new cases of autoantibody positivity appeared after RAI. On the whole, the available data seem to indicate that early hypothyroidism after rhTSH pretreatment is more frequent than after RAI alone, irrespective of rhTSH dosages and RAI activities. However, the possibility cannot be ruled out that the difference observed is due, at least in part, to differences in baseline thyroid function between the two groups of patients.

Elderly patients with large MNG may have underlying cardiovascular disease, and rhTSH and RAI administration may engender a risk of cardiac complications, owing to the increase in thyroid hormones. In a recent report by Barca et al. [29] on patients aged 42 – 80 years who were treated with a fixed 1100 MBq activity of RAI after 0.1 mg of rhTSH, a 3-fold increase in left ventricular diastolic dysfunction was seen in subjects with laboratory evidence of hyperthyroidism. This [29], as well as other previous studies [11], indicates that beta-blockers should be routinely administered to reduce thyrotoxic symptoms when rhTSH and RAI therapy are administered. In our protocol, at least one daily 25 mg dose of atenolol was administered by default; only one patient from the rhTSH and RAI group suffered a severe, albeit transient, cardiovascular adverse event, while in the RAI group an increase in pre-existing dyspnea was sometimes reported. This indicates that, in selected elderly subjects, the adjuvant administration of rhTSH does not worsen RAI-induced cardiovascular adverse events. On the other hand, in our patients the simultaneous administration of anti-inflammatory steroids resulted in less symptomatic swelling of thyroid tissue, a condition which has been reported by 10% to 30% of patients after rhTSH and RAI [18].

On the basis of the present and previous studies, rhTSH pre-stimulation can improve the goiter reduction achieved by RAI therapy. Administering small doses of RAI and rhTSH can limit side-effects without compromising the goiter-reducing effect, while improving MNG-related symptoms in elderly subjects. RhTSH-induced ^131-^I uptake in less active thyroid areas of the goiter, and perhaps rhTSH-induced mechanisms other than the increase in thyroid uptake, could explain the better results achieved by using rhTSH as an adjunct to RAI therapy.

## Competing interests

The authors declare no conflict of interest. This study was partially supported by a grant from the University of Genoa (Finanziamento Progetti di Ateneo 2007).

## Authors' contributions

MG conceived the study, performed the statistical analysis and drafted the manuscript. MC participated in the design of the study. IC participated in the design of the study. MB carried out the instrumental evaluations. EC carried out the assays. MS participated in the design of the study. MM participated in the coordination of the laboratory data. DF participated in the sequence alignment. FM participated in the conception of the study. MB conceived the study and drafted the manuscript. All authors have read and approved the final manuscript.
